# Longitudinal analysis of circulating tumor cell numbers improves tracking metastatic breast cancer progression

**DOI:** 10.1038/s41598-024-63679-4

**Published:** 2024-06-05

**Authors:** Malgorzata Szostakowska-Rodzos, Anna Fabisiewicz, Maciej Wakula, Sylwia Tabor, Lukasz Szafron, Agnieszka Jagiello-Gruszfeld, Ewa Anna Grzybowska

**Affiliations:** Maria Sklodowska-Curie National Research Institute of Oncology, Warsaw, Poland

**Keywords:** Circulating tumor cells, Liquid biopsy, Metastatic breast cancer, Hormone-responsive breast cancer, Estrogen resistance, Breast cancer, Cancer genetics, Metastasis, Tumour biomarkers, Tumour heterogeneity

## Abstract

Hormone-responsive breast cancer represents the most common type and has the best prognosis, but still approximately 40% of patients with this type can develop distant metastases, dramatically worsening the patient’s survival. Monitoring metastatic breast cancer (mBC) for signs of progression is an important part of disease management. Circulating tumor cell (CTC) detection and molecular characteristics gain importance as a diagnostic tool, but do not represent a clinical standard and its value as a predictor of progression is not yet established. The main objective of this study was to estimate the prognostic value of not only the CTC numbers, but also the dynamics of the CTC numbers in the same patient during the continuous evaluation of CTCs in patients with advanced breast cancer. The other objective was to assess the molecular changes in CTCs compared to primary tumor samples by genetic analysis of the seven genes associated with estrogen signaling pathway, mutations in which are often responsible for the resistance to endocrine therapy, and subsequent progression. This approach was taken to evaluate if genetic analysis of CTCs can be used in tracking the resistance, signaling that hormonal therapy should be replaced. Consequently, this report presents the results of a longitudinal CTC study based on three subsequent blood collections from 135 patients with metastatic breast cancer, followed by molecular analysis of the isolated single CTCs. CTCs were detected and isolated using an image-based, EpCAM-independent system CytoTrack; this approach allowed evaluation of EpCAM expression in detected CTCs. Isolated CTCs were subjected to NGS analysis to assess mutational changes. The results confirm the importance of the status of the CTC for progression-free survival and overall survival and provide new data on the dynamics of the CTC during a long monitoring period and in relation to clinical progression, highlighting the advantage of constant monitoring over the single count of CTC. Furthermore, high genetic and phenotypic inter- and intrapatient heterogeneity observed in CTCs suggest that metastatic lesions are divergent. High genetic heterogeneity in the matching CTC/primary tumor samples may indicate early dissemination. The tendency towards the accumulation of activating/oncogenic mutation in CTCs, leading to anti-estrogen resistant disease, was not confirmed in this study.

## Introduction

Breast cancer (BC) is the most frequently diagnosed cancer and is the second leading cause of cancer-related deaths in women worldwide. Breast cancer is a heterogeneous disease that comprises tumors with various genomic and clinical characteristics. Luminal cancers (expressing estrogen and progesterone receptors) represent the majority of diagnosed breast cancers, with a frequency of 50–80%^1,2^. Despite the use of targeted anti-estrogen therapies, approximately 30–50% of patients with ER-positive breast cancer will relapse due to resistance to the given treatment. As repeatedly shown, the status of the estrogen receptor (ER) may differ between primary tumor and metastatic lesions^3,4^, pointing to mutations as a source of resistance.

Enumeration of CTC in metastatic breast cancer is very well characterized as a prognostic and predictive factor^5–7^. In mBC studies the cutoff of ≥ 5 CTCs is widely accepted as significant for prognosis^8^. However, most of the existing reports describe the results of a single blood collection or an initial blood collection at the beginning of treatment with only one follow-up. Some reports describe the analysis of serially collected CTC^9–12^, but more data are needed to establish the clinical value of monitoring the number of CTC in patients with mBC. Additionally, most existing reports describe the results obtained using EpCAM-dependent Cell Search system, potentially omitting EpCAM-low and negative CTCs.

Molecular characterization of CTCs, in addition to simple enumeration, offers an expanded ability to monitor and control the disease by tracking mutational changes in ERα and other genes involved in the estrogen signaling pathway or associated pathways^13^. In patients with mBC, molecular analysis of CTC can be a proxy for metastatic lesion biopsy and serve as a monitoring system for changes in ER status, contributing to the proper evaluation of the disease and possible treatment decisions about abandoning anti-estrogen therapy and including chemotherapy.

In this report, we describe the analysis of CTC in three subsequent blood collections and an initial estimate of the potential clinical value of longitudinal monitoring of CTC in patients with mBC. The analysis was performed using an EpCAM-independent system, which enables to estimate EpCAM expression in detected CTCs and include EpCAM-negative CTCs. The study contains genetic analysis of isolated single CTCs and a comparison of their mutational status with primary tumor samples. The analysis encompassed seven genes associated with estrogen signaling pathway, often mutated in breast cancer, leading to hormone-resistant disease and cancer progression. This analysis aimed to track the development of the estrogen resistance on the way to a secondary site, in the CTCs in the bloodstream and/or to track the genetic changes in the secondary site without biopsy, assuming that CTCs in the circulation are derived from the metastatic lesions.

The results confirm > 5 CTC numbers as prognostic, but also highlight the superiority of constant monitoring over a single analysis, especially in the case of persistently high or constantly increasing CTC count. Furthermore, the results accentuate the high phenotypic and genetic heterogeneity of isolated CTC, as well as primary tumor in luminal BC.

## Materials and methods

### Patients

**Figure 1:**
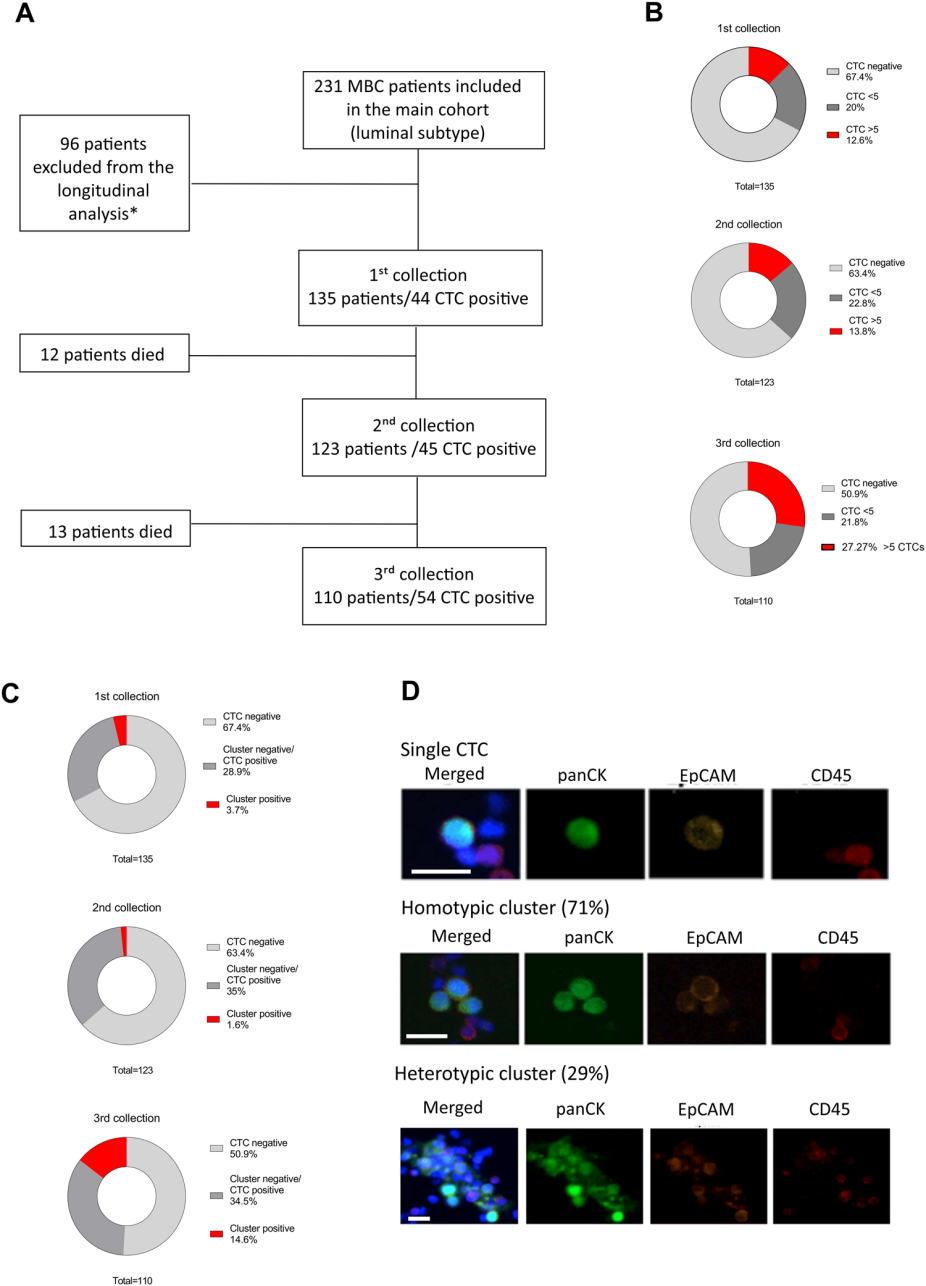
Flow chart of the study cohort and CTCs enumeration data. (**A**) Study cohort and time points for longitudinal CTC analysis. *exclusion criteria: insufficient clinical data or quality of CTC sample (**B**). CTC numbers at all three time points categorized as none, < 5, > 5 CTCs. (**C**) Proportion of CTC clusters detected at all three time points in relation to single CTCs. (**D**) Types of single CTCs and CTC clusters detected during analysis. Homotypic clusters are predominant (71%), but heterotypic clusters containing immune cells (CD45 staining) are also present. Staining: tumor markers (pan-cytokeratin—CK, EpCAM), WBC (CD45), nuclei (DAPI). Images generated using the CytoTrack system.

In total, 237 patients with diagnosed luminal mBC were enrolled in the study between June 2018 and October 2020. The selection was carried out in cooperation with experienced clinicians from the Department of Breast Cancer and Reconstructive Surgery of the National Research Institute of Oncology. The study protocol was approved by the Ethics Committee of the National Cancer Research Institute (34/2016). The inclusion criteria for the patients: breast cancer with ongoing hormonal treatment, age > 18 and the identification of distant metastases. All participants signed an informed consent. Blood collection was carried out three times during treatment in 3-month intervals. In general, 539 samples were collected from 237 patients, but some patients were excluded due to not sufficient quality of the samples, not acceptable intervals between collections, or incomplete medical record, leaving 135 patients included in the final analysis (Fig. 1A).

### CTC detection/isolation (CytoTrack)

Detection was carried out using CytoTrack, a method without enrichment-associated bias^14,15^.

#### Sample processing

Blood samples (9 ml) were collected in EDTA tubes and processed up to 2 h after collection. The samples were centrifuged at 2500×*g* for 15 min. The buffy coat containing nuclear cells, including tumor cells, was transferred to a new 15 ml tube. The residual erythrocytes were lysed twice with FACS Lysing solution (BD Biosciences) and with incubation times of 15 and 10 min at room temperature. The samples were centrifuged at 3000×*g* for 5 min. The cells were permeabilized with permeabilization buffer (1xPBS, 0.25% Saponin, 0.5% BSA) for 15 min RT and centrifuged at 3000×*g* for 5 min. Permeabilized cells were stained with: Alexa Fluor 488-conjugated pancytokeratin (pan-CK) antibody (1:25) (ThermoFisher Scientific), APC conjugated CD45 antibody (1:10) (ThermoFisher Scientific), PE conjugated EpCAM antibody (1:10) (ThermoFisher Scientific), and 4,6-diamidino-2-phenylindole (DAPI)(Sigma) (1:1000). Incubation time: 60 min, 4 °C in the dark. Stained cells were washed twice with blocking buffer (1xPBS, 1% BSA), centrifuged at 3000×*g* for 5 min, resuspended in 1ml of ultra-pure H_2_O, smeared on the glass disc (CytoDisc) and dried overnight. Dry samples were mounted with CytoCover and fixed with the use of fixogum. The samples were stored at -80°C until CytoTrack scanning.

#### Scanning and CTC detection

For scanning, a focus plan was obtained based on the DAPI channel, at eight places on the disc. Scanning was performed with a 488 nm argon-neon laser, in a spiral pattern with a bandwidth of 10 μm, for 5 min. All signals from the Alexa Fluor 488 emission channel were recorded and listed in the hotspot table (positive events). Criteria for CTC identification were established as: nearly round size with ≥ 6µm diameter, visible nucleus, pan-CK signal, CD45 negative. Clusters were defined as: group of ≥ 3 cells, with at least 3 visible nuclei in the DAPI channel, with at least 3 cells identified as CTCs. Homotypic clusters were defined as clusters composed only of cancer cells. Heterotypic clusters were defined as clusters composed of both PBMC and cancer cells. All samples were scanned and cells that met the CTC criteria were counted. For identified CTCs, sets of images were taken for every fluorescent channel for further EpCAM expression analysis. All coordinates of the identified CTCs were saved for micromanipulation.

#### Cell picking and whole genome amplification (WGA)

Single CTCs were isolated from the discs by CytoPicker (micromanipulator). The cover glasses of the discs were detached by overnight incubation in 1xPBS buffer and the discs were left for air-drying. The CTCs were retraced using saved coordinates. Cells were picked individually in a maximum 5 µl volume of 1xPBS buffer. The selected single cells were transferred to PCR tubes and whole genome amplification was performed with the MALBAC WGA kit (YikonGenomics), according to the manufacturer’s instructions. Single cell WGA products were visualized on 1% agarose gel and analyzed on Bioanalyzer (Agilent Technologies) with the use of High Sensitivity DNA kit (Agilent Technologies). Only WGA products with distribution lengths between 200 and 3000 bp were chosen for next-generation sequencing (NGS).

### NGS analysis

The NGS was performed using a custom library designed in Illumina Design Studio (Illumina). The amplification sequencing method was chosen. The designed library covered the main hotspots in the *ESR1* (exons: 4, 5, 8), *PIK3CA* (exons: 9, 20) and *AKT1* (exon 4) genes and all coding regions for the genes: *TP53*, *GATA3*, *ESR2*, *AKT2*. The coverage of the amplicons that build the library was 99.84%. The total size of the library was 8368 bp and the average amplicon length was 136 bp. The designed library contained two pools of primers, with 70 primer pairs per pool. Libraries were prepared using Illumina Library PLUS (Illumina) according to the manufacturer’s instructions. The libraries were then quantified via Quantus (Promega) using QuantiFluor dsDNA ONE system (Promega), purity and size of the libraries were established using High Sensitivity DNA kit (Agilent Technologies). As libraries were prepared with the use of WGA product as a template, in some libraries the WGA artifacts were detected. Libraries with WGA artifacts were additionally purified using a BluePipin (Sage Science) system. Libraries after BluePipin purification were measured using Quantus and Bioanalyzer, to confirm the elimination of WGA artifacts (Fig. S1). Libraries were sequenced using the MiniSeq Mid-Output Kit (Illumina). Sequencing depth was set as × 300 with bases higher than Phred quality score of Q30 for about 95% of reads. Sequencing data were analyzed and plotted using maftools (MAF: Mutation Annotation Format)^16^. For each sample, the reads were extracted from the original BAM file using the cell-specific barcodes and were aggregated to generate a sub-BAM file. Mutect2 (v.4.1.0.0) was then applied to the sub-BAM files to identify somatic point variants. Then, outputs were run through pipeline for filtering and annotation. Variant allele frequency (VAF) < 0.2 was discarded, unless they were present in the primary tumor sample. Heterogeneity was calculated as the frequency of common variants (detected in many cells) and unique variants (detected in one cell). The number of common variants was indicated in %, in formula: no of common variants/no of all detected variants × 100%, or for unique variants: no of unique variants/no of all detected variants × 100%. Pathogenicity was estimated using CADD algorithm^17^. VCF files from NGS analysis were reformatted and analyzed using CADD v1.7 version. Raw CADD score was calculated and plotted to rank deleteriousness of genetic variants.

### FFPE analysis

FFPE samples obtained from NIO-PIB Department of Cancer Pathomorphology were cut 10 µm thin and up to 8 sections were used for DNA isolation. DNA was isolated using the QIAamp DNA FFPE Tissue Kit (Qiagen) according to the manufacturer’s instructions. The purity of the isolated genetic material was verified using a NanoDrop spectrophotometer (ThermoFisher). Only pure DNA with a concentration of at least 50 ng/µl were used for sequencing. Samples were amplified using GoldTaq Polymerase (Applied Biosystems) and GeneAmp PCR System 9700 Thermal Cycler (Applied Biosystems). The conditions of the amplification reactions are presented in Tables S1 and S2. PCR products were further sequenced using BigDye™ Terminator v3.1 Cycle Sequencing Kit (Thermo-Fisher) and ABI Prism 3130xl Genetic Analyzer.

### EpCAM expression

In total, 2649 images of 64 patients were documented (from 90 Cytodiscs). For the analysis, 2542 images were selected from 31 patients (41 Cytodiscs). Only samples with more than 5 good quality images were analyzed. Low quality images or high background noise images were excluded from the analysis as Otsu’s threshold clustering was unable to calculate proper ROI area. ROC analysis was performed for the EpCAM/panCK ratio, with values from the MCF7 cell line (ATCC) as the control group.

### Statistical analysis

Categorized quantitative data at different time points were compared using the Mann–Whitney U test or, if there were more than two categories, the Kruskal–Wallis test. The primary end point was overall survival (OS), the secondary end points were progression-free survival (PFS) and progression versus nonprogression in relation to CTCs numbers/presence and the CTCs dynamics. Progression during observation time was defined as progression sets using magnetic resonance imaging and/or computed tomography. If the patient progressed for a few imaging sets in a row, it was treated as a one-long progression. The new progression was defined as progression after achieving stable disease in the imaging sets. The time from the date of blood collection to progression or death from any cause was calculated. If an outcome was not reached during the observation time, the variables were censored. Kaplan–Meier plots and the log-rank tests were used to illustrate and compare survival between subgroups. Survival analysis of variables measured at follow-up collections was performed using landmark analysis. Univariable and multivariable hazard ratios (HR) for selected potential predictors of PFS and OS were determined by Cox proportional hazards regression. Model fit was measured using the Harrell C index, and the fit of nested prognostic models was compared using the log-likelihood ratio (G squared) test. The association between CTC presence and dynamics with primary and secondary end points was also tested by logistic regression. All data were analyzed using GraphPad Prism 9.

### Ethics approval

The study protocol was approved by the Ethics Committee of the National Cancer Research Institute (34/2016). All subjects with a confirmed diagnosis of mBC agreed to participate according to the ethical guidelines of the 2013 Declaration of Helsinki. The material was anonymized at the time of collection.

## Results

### Patients and study design

The 135 patients were enrolled in the longitudinal analysis with three subsequent blood collections (one collection every three months, to match the structured clinical and radiological evaluation). Patient and tumor characteristics are summarized in Table 1. The median follow-up time from the first collection was 20.93 months (range 1–25) for patients alive at the last visit before the cutoff date of 31 July 2022. 35 patients (23.7%) had one metasite at the beginning of observation (bone metastases only), 43.9% of the patients were identified with two distant metasites, and 33.3% of the patients with 3 distant metasites. Most of the patients were treated with hormonal treatment combined with chemotherapy (HTH + CHTH). Only 12.5% of the patients were treated with CDK4/6 inhibitors combined with hormonal therapy (HTH + CDK4/6 inhibitors) and 25.18% of the patients were treated only with hormonal therapy (HTH). The flow chart of the study cohort is shown in Fig. 1A.

**Table 1:** The clinicopathological characterization of the group of patients enrolled in the study.

Clinical feature	No. of patients
Age at the 1st blood collection	
< 65	78
≥ 65	57
HER2 status	
HER2−	132
HER2+	3
TNM	
TxN0M0	35
TxNxM0	51
TxNxMx	49
No. of distant metastases sites	
1	32
2	58
≥ 3	45
Metastatic sites	
Bones	114
Liver	43
Lungs	40
Other	57
Treatment during study	
HTH + CHTCH	84
HTH	34
HTH + CDK4/6inh	17
Histological type	
NST (ductal)	103
Lobular	17
Ductal-lobular	6
Other	9
BRCA1/2	
WT	129
Mutant	6

### Clinical value of CTC detection in longitudinal study

#### CTC count

CTCs were detected in 143 samples collected from 90 patients during the observation time. CTCs were detected in 44 (32.59%) patients in the first blood collection, 45 (36.58%) patients in the second blood collection, and 54 (49.09%) patients in the third blood collection. Moreover, ≥ 5 CTCs were detected in 17 patients in the first and second blood collection (12.59% and 13.82%, respectively) and in 30 (27.27%) patients in the third blood collection (Fig. 1B). The clusters were detected in the material collected from 23 patients and some fraction of the clusters was present in all three collections (Fig. 1C). In general, we identified over 160 clusters in all samples, mostly homotypic (composed of tumor cells, 71%) with a heterotypic (composed of tumor cells and immune cells) cluster fraction of 29% (Fig. 1D). However, since clusters were observed mainly in the last blood collection, the presence of clusters was not further statistically analyzed.

### Survival analysis confirms the importance of more than 5 CTCs in the sample

**Figure 2:**
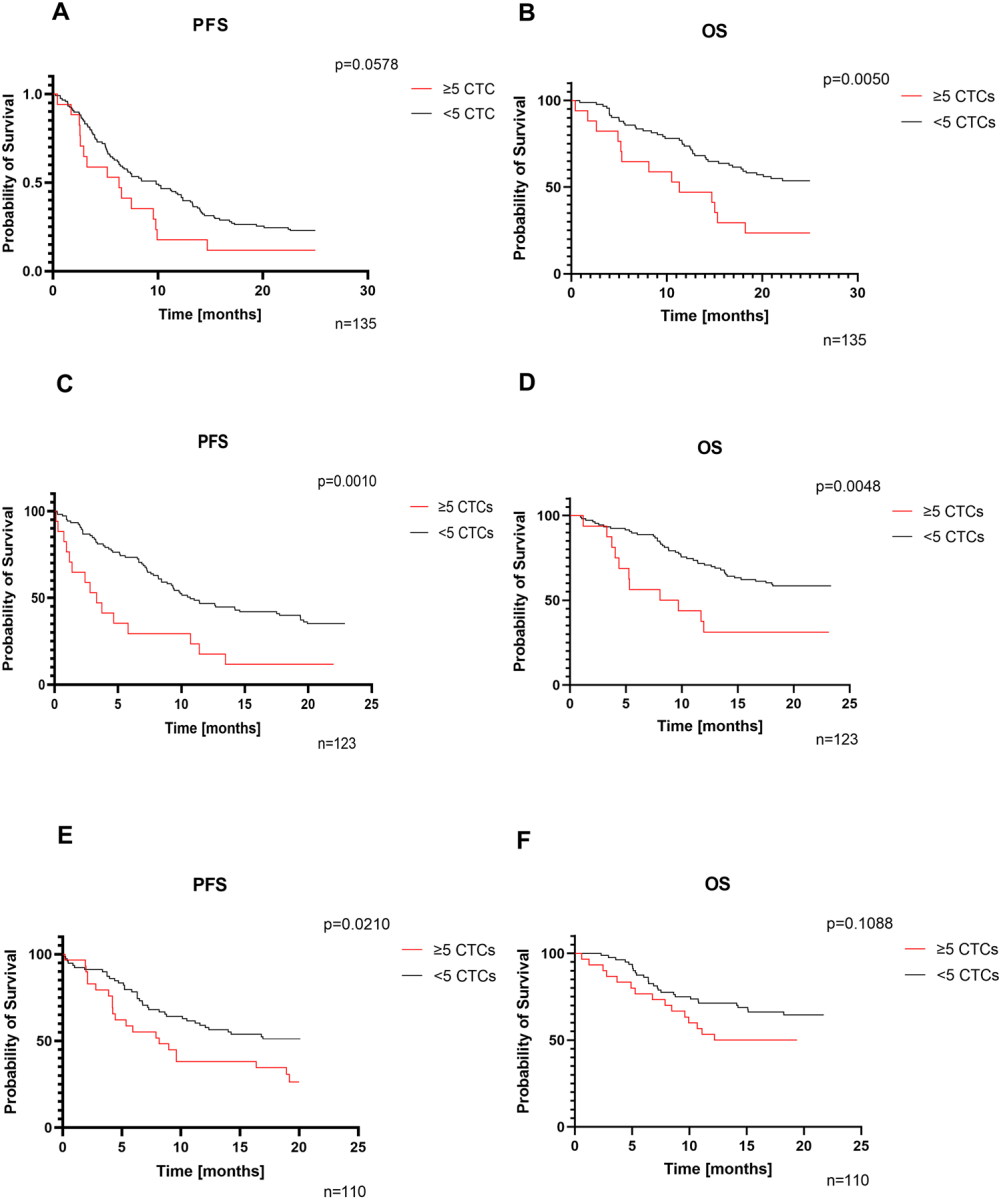
Kaplan-Mayer survival curves with p-value from log-rank tests for ≥ 5 or < 5 CTC. (**A**, **B**) Data from the first collection landmark; (**C**, **D**) data from the second collection landmark; (**E**, **F**) data from the third collection landmark. The mean survival (in months) was calculated only for groups where survival reached 50% during the observation time.

Kaplan–Meier survival analysis and landmark analysis for follow-up collections indicated that although CTCs presence/absence in the first collection was not significant for PFS and OS, detection of ≥ 5 CTCs was identified to be significant for OS (log-rank test p < 0.05) and a strong trend for PFS was also observed (log-rank test p = 0.0578) (Fig. 2A,B). In the second collection, the identification of ≥ 5 CTCs was found to be a strong predictor of PFS and OS (log-rank test p < 0.05) (Fig. 2C,D). In the third collection, the presence of ≥ 5CTCs was a significant predictor only for PFS (log-rank test p < 0.05) (Fig. 2E,F). As the third blood collection was performed in the most advanced group of patients, the value of ≥ 5CTCs for PFS and OS was verified with the Gehan-Breslow-Wilcoxon test, which gives more weight to events/deaths at early time points. Statistical significance was still observed for PFS (p-value = 0.0314), but not for OS (p-value p = 0.0873).

#### CTC dynamics in serially collected samples

**Figure 3:**
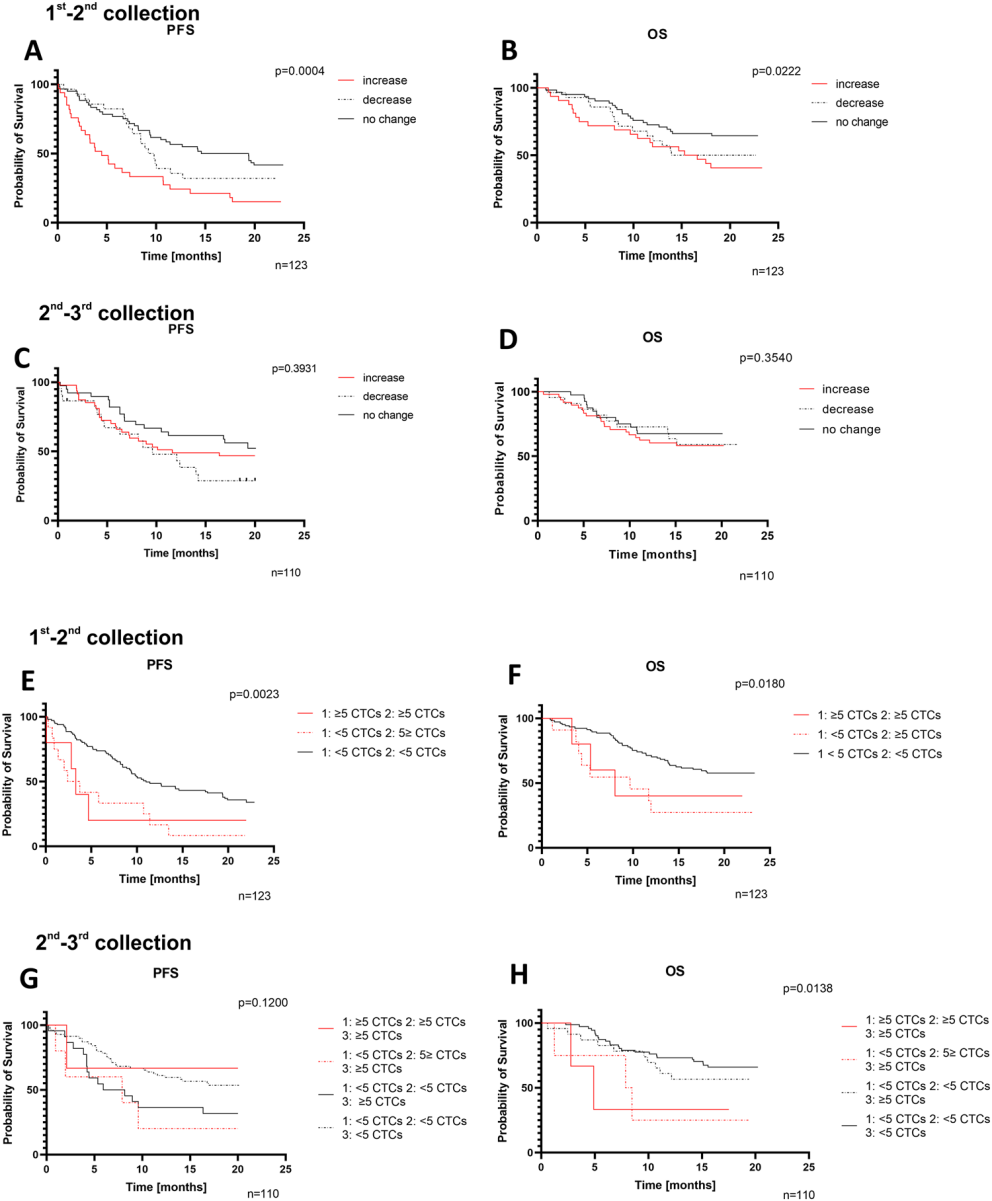
The Kaplan–Mayer analysis of changes in CTCs numbers between collection landmarks with p-values from log-rank tests. The patients were divided into subgroups according to the CTC counts in every blood collection. (**A**–**D**) Dynamics of changes (increase, decrease, no change) (**E**–**H**). Number of CTCs (≥ 5 or < 5) in the sample.

An analysis of CTC dynamics after second (changes between 1st and 2nd) and third (changes between 2nd and 3rd) blood collections was performed, with tracking subsequent CTC count in each patient. The patients were divided into groups with increased, decreased, and no change in the number of CTC (Fig. 3A–D). For the second blood collection, dynamics was a significant predictor of poorer PFS and OS (log-rank p-value < 0.05). Patients with an increase in CTC count in the second blood collection were characterized with shorter median survival for PFS (4.47 months) and OS (15.97 months) than patients with a decrease or no change in CTC numbers (PFS-9.5 months, OS-18.3 months and PFS-16.985 months, OS-undefined, respectively, Fig. 3A,B). In the third collection, an increasing number of CTCs was a poorer predictor of PFS and OS than in the second collection (Fig. 3C,D). All median OS and PFS for groups are provided in Supplemental Table S3.

Additionally, for the second blood collection constant high CTC counts (≥ 5 CTCs) and high CTC (≥ 5 CTCs) count in the second, but not in the first blood collection, were identified as significant unfavorable predictors of PFS and OS (Fig. 3E,F). Patients with constant high CTC counts and high CTC counts from the second collection were identified with shorter median survival for OS (4.9 months and 8.17 months, respectively). For patients with high CTC counts only in the third blood collection and patients with constant low CTC numbers (< 5 CTCs) the median survival for OS was not defined (Fig. 3E–H). The group with < 5 CTCs in all collections was considered a reference group.

#### CTC as predictors of survival in Cox proportional hazard regression models

##### CTC count

**Figure 4:**
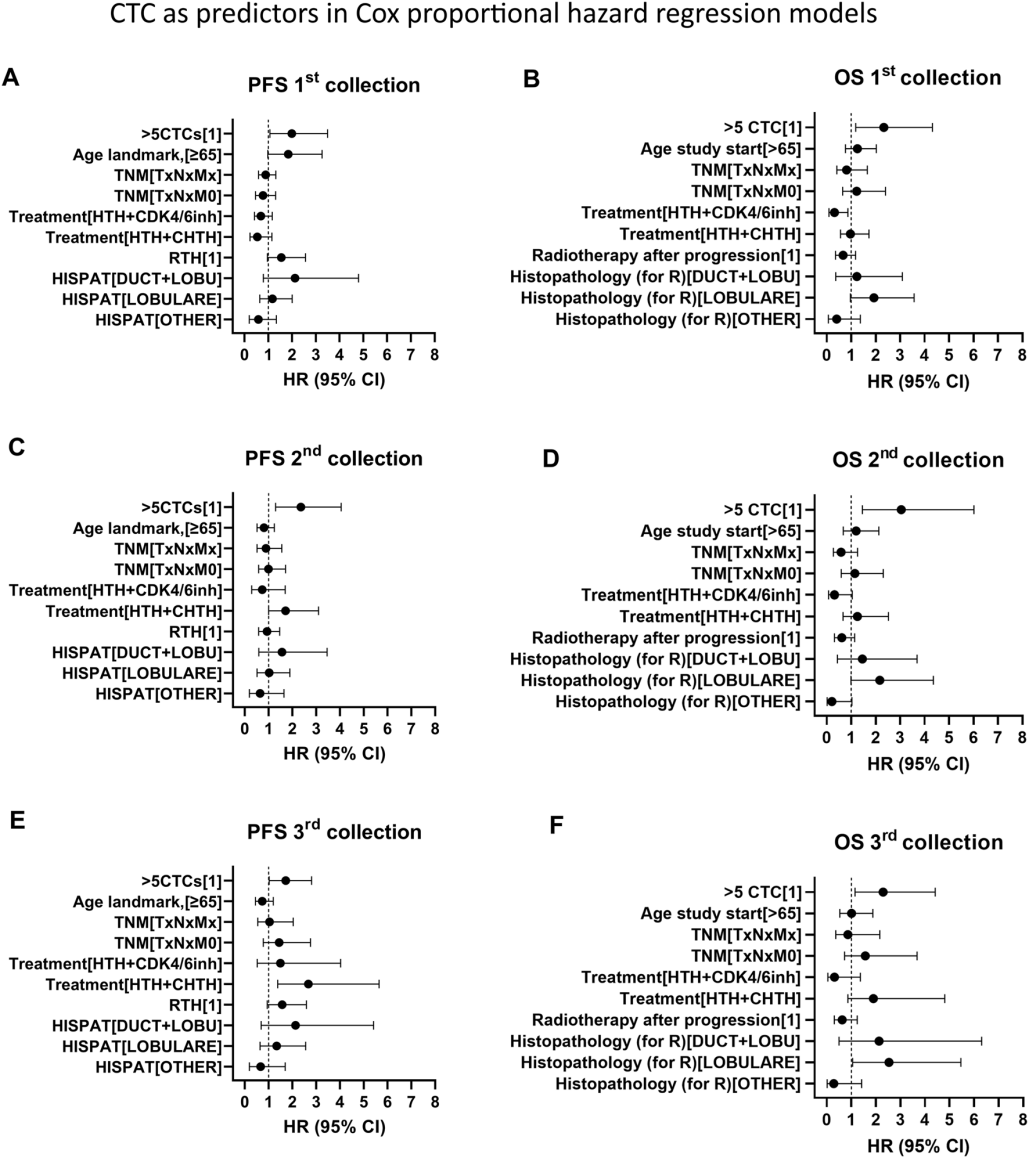
The hazard ratio (HR) of predictors used in multivariable Cox proportional hazard regression for PFS and OS with the 95 confidence intervals. (**A**, **B**) The results of the multivariable COX proportional hazard regression analysis for ≥ 5CTCs, for the first collection landmark. (**C**, **D**) The results of the multivariable COX proportional hazard regression analysis for ≥ 5CTCs, for the second collection landmark. (**E**, **F**) The results of the multivariable COX proportional hazard regression analysis for ≥ 5CTCs, for the third collection landmark.

Univariable and multivariable Cox proportional hazard regression models were built to analyze the data. The data used for the Cox hazard regression were categorical variables with the reference level defined in the model. The covariates used for the multivariable clinicopathological model were: histopatological type, RTH during observation, TNM status at the time of diagnosis, type of treatment, and age. All clinicopathological data with their reference levels are listed in Table S4. In the univariable analysis, the presence of ≥ 5CTCs in the first blood collection was found to be a strong predictor of death (HR 2.3 95% CI 1.201–4.083, p = 0.0071), but not of PFS. In the multivariable model, the high count of CTC in the first collection was identified as a strong predictor of both, OS (HR_OS_ 2.323) and PFS (HR_PFS_ 1.987), (Fig. 4A,B). In the second blood collection the presence of ≥ 5CTCs was also a strong predictor of OS and PFS in univariable (HR_OS_ 2.724 and HR_PFS_ 2.539) and multivariable (HR_OS_ 3.004; 95% CI 1.453–6.016; p-value < 0.05 and HR_PFS_ = 2.359) analyzes (Fig. 4C,D). In the third collection, the high CTC count (≥ 5CTCs) was not a significant predictor in the univariable analysis, but it was found to be predictive of OS (HR_OS_ = 2.29) and PFS (HR_PFS_ = 1.72) in the multivariable analysis (Fig. 4E,F). The results of the analysis are summarized in Table 2, Table S5 and Fig. 4. In general, we found that in the presented clinicopathological model the ≥ 5CTCs count is a strong predictor of death and progression, regardless of the blood collection time point.

**Table 2:** The results of Cox proportional hazard regression models showing the most significant parameters (values for all parameters in Table S4).

Variable	OS			PFS		
	HR	95 CI	p-value	HR	95 CI	p-value
1st collection						
Histopatological [Lobulare]	1.929	0.9650–3.570	0.047	1.177	0.6457–2.008	ns
> 5 CTCs	2.323	1.175–4.320	0.0105	1.987	1.067–3.493	0.0223
Treatment [HTH + CDK4/6i)	0.3131	0.08908–0.8579	0.0391	0.5364	0.2308–1.148	ns
2nd collection						
Histopatological [Lobulare]	2.167	0.9877–4.358	0.0392	1.025	0.5176–1.892	ns
> 5 CTCs	3.044	1.453–6.016	0.002	2.359	1.296–4.051	0.003
CTCs dynamics						
Histopatological [Lobulare]	2.1485	0.9669–4.306	0.04	1.058	0.5362–1.946	ns
	2.173	1.002–4.306	0.0351	1.054	0.5387–1.918	ns
Increase in 2nd collection to > 5 CTCs	3.067	1.343–6.348	0.0042	2.261	1.149–4.100	0.0112
< 5CTCs	0.3338	0.1798–0.6334	0.0006	0.4423	0.2717–0.7429	0.0014
3rd collection						
> 5 CTCs in collection						
Histopatological [Lobulare]	2.53	1.054–5.465	0.025	1.339	0.6449–2.556	ns
Treatment [HTH + CHTH]	1.895	0.8595–4.805	ns	2.671	1.386–5.641	0.0057
> 5 CTCs	2.29	1.145–4.417	0.0154	1.72	1.027–2.804	0.0336
CTCs dynamics						
Histopatological [Lobulare]	2.693	1.123–5.798	0.0165	1.381	0.6627–2.647	ns
Treatment [HTH + CHTH]	1.655	0.7384–4.228	ns	2.53	1.313–5.326	0.0088
< 5CTCs	0.4434	0.2246–0.8725	0.0181	0.5867	0.3671–0.9468	0.0267

##### CTC dynamics

To investigate the potential value of the changes in CTC numbers during treatment, we also analyzed the survival data from the second blood collection and the third blood collection landmarks with respect to specific CTC dynamics. We confirmed that increasing CTC counts in the second blood collection are unfavorable predictors of OS and PFS. Patients with CTCs counts that increased to ≥ 5 CTCs were characterized with 2.933 (95% CI 1.340–5.723; p-value < 0.05) times higher risk of death than patients with other dynamics in univariable analysis. Furthermore, in univariable analysis, patients with this dynamic were also characterized with 2.516 (95% CI 1.298–4.457; p-value < 0.05) times higher risk of progression than patients with > 5CTCs only in 3rd collection or with constant low CTC counts. The strong predictive value of this dynamic remains significant in multivariable analysis (HR_OS_ 3.067; 95% CI 1.343–6.348; p-value < 0.05 and HR_PFS_ 2.261; 95% CI 1.149–4.100; p-value < 0.05) (Table 2).

We also confirmed the prognostic value of persistent high counts of CTCs. In the univariable analysis patients with continual ≥ 5CTCs in the second and third blood collection were characterized with a risk of death 3.728 times higher (95% CI 1.112–9.346; p-value < 0.05) than patients with other dynamics. In multivariable analysis, the hazard ratio for OS increased to 7.001 (95% CI 1.744–23.33; p-value < 0.05) (Table 2). However, this factor was not found to be a significant predictor of PFS. Additionally, we observed that a constant low number of CTCs (< 5 CTCs in all collections) is a strong favorable predictor for both OS and PFS in all analyses (Table 2).

##### CTC count as a predictor of progression

During the total observation time (9 months), 29 patients did not progress and 105 patients had clinical signs of progression.

To evaluate the CTC count as a predictor of rapid progression, the number of CTC and the dynamics of the CTC were analyzed in relation to the clinical outcome (death or progression) that occurred within 3 months of collection. In logistic regression models the patients with ≥ 5 CTCs detected in the first blood collection had significantly higher mortality odds (OR 4.231; 95% CI 1.019–15.55; p-value < 0.05) than patients with < 5 CTCs detected. In the second collection for patients with ≥ 5 CTCs overall survival was not significantly different, but they had significantly higher odds of progression occurring 3 months after collection (OR 3.273; 95% CI 1.088–9.916; p-value < 0.05) than patients with < 5 CTCs. In the third blood collection patients with ≥ 5 CTCs were characterized by significantly higher odds of mortality and odds of progression occurring in 3 months after collection than patients with < 5 CTCs (Table S6).

Interestingly, patients with CTC numbers that continuously increased in each blood collection were characterized by higher odds of progression/death occurring within 3 months after the third blood collection than patients with other dynamics (OR 8.091; 95% CI 1.976–33.75; p-value < 0.05) (Table S6).

### Phenotypic and genetic heterogeneity of isolated CTCs

#### EpCAM expression in analyzed CTCs displays temporal, inter- and intra-patient heterogeneity

**Figure 5:**
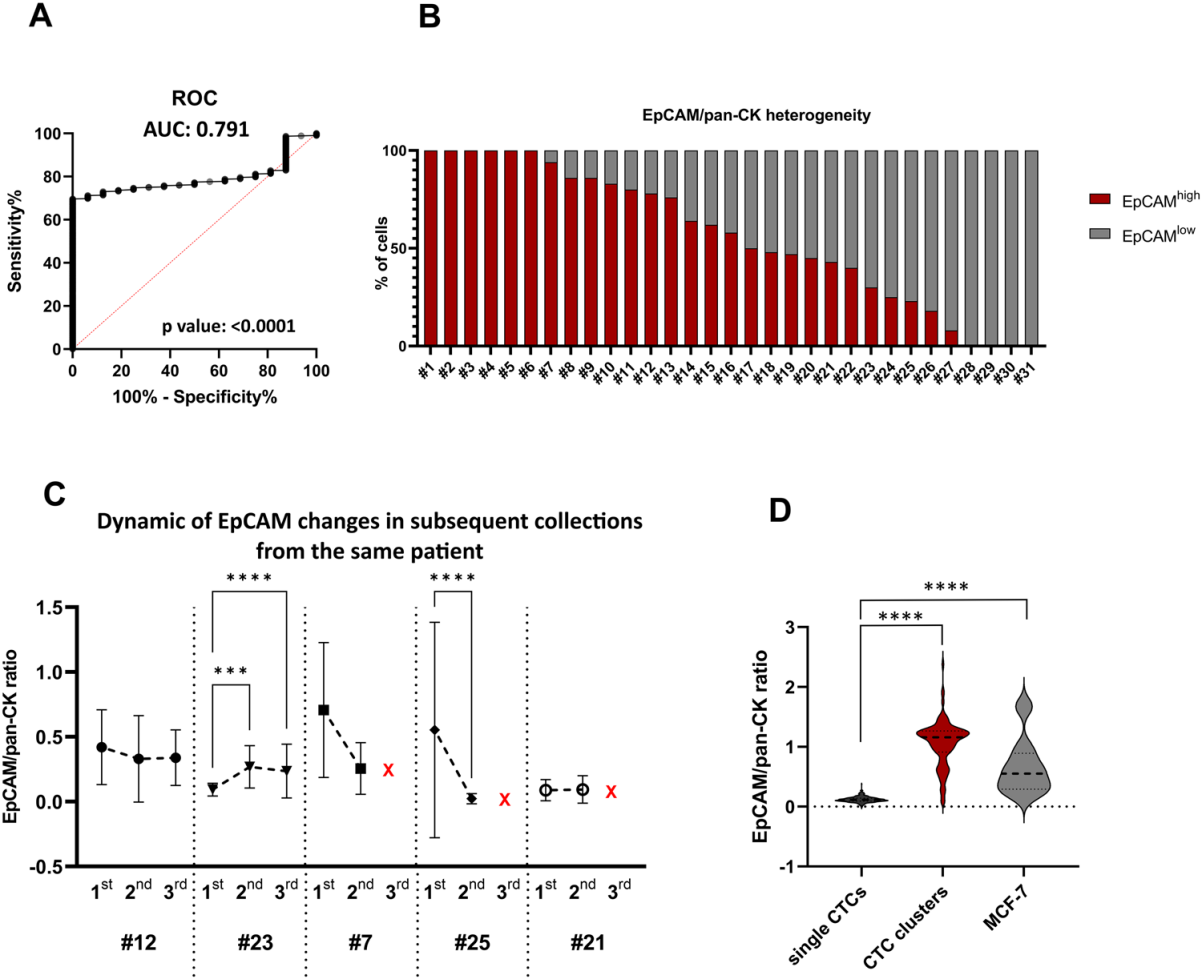
EpCAM/pan-CK heterogeneity among patients. (**A**) ROC curve to establish the EpCAM/pan-CK ratio cut-off value to classify cells as EpCAM^high/low^. Area under the curve: 0.7910, SD = 0.02448; p-value < 0.0001. The data from MCF-7 single cells was used as a reference measurement (control). The cut-off value was set to 0.2213, according to the highest LikelihoodRatio (GraphPad9). (**B**) The percentage of CTCs with different EpCAM/pan-CK ratios between patients. EpCAM^low^ represents the EpCAM/pan-CK ratio lower than 0.2213; EpCAM^high^ represents the EpCAM/pan-CK ratio higher than 0.2213. (**C**) Dynamic changes of the EpCAM/pan-CK ratio in subsequent collections from the same patient. The red ‘X’ marks the death of the patient. Error bars: SD. (**D**) Representation of the total EpCAM/pan-CK ratio calculated for CTC in patient samples separately for single CTCs and CTC clusters. The ratio in cells of the MCF-7 cell line (spiked blood from a healthy donor) was used as a reference.

The CytoTrack System is EpCAM independent, therefore it enables the analysis of EpCAM-positive and EpCAM-negative CTCs. To further explore this possibility, inter- and intra-patient EpCAM heterogeneity was analyzed. In total, 2542 images from 31 patients were analyzed. Only samples with more than five good quality images were selected for the analysis. EpCAM expression was evaluated as an EpCAM/panCK ratio, with a cut-off value of 0.2213 established in the ROC analysis (Fig. 5A) to distinguish the EpCAM^low^ and EpCAM^high^ groups. The EpCAM^high^ phenotype was observed in 56% of the analyzed CTCs, EpCAM^low^ in 44%, while a complete EpCAM negative phenotype was detected in 0.5% of the CTCs (EpCAM^low^ and negative CTCs are potentially omitted in the CellSearch analysis). The results indicate that the expression of EpCAM in CTC varies between patients (Fig. 5B), but also varies in the same patient during treatment (Fig. 5C), and the changes in the same patient are not unidirectional. Interestingly, the EpCAM/panCK ratio was significantly higher in CTC clusters, compared to the single CTCs and to the reference MCF7 cell line, while most of the single CTCs have a lower EpCAM/panCK ratio than in the reference cell line (Fig. 5D).

#### Genetic interpatient heterogeneity

**Figure 6:**
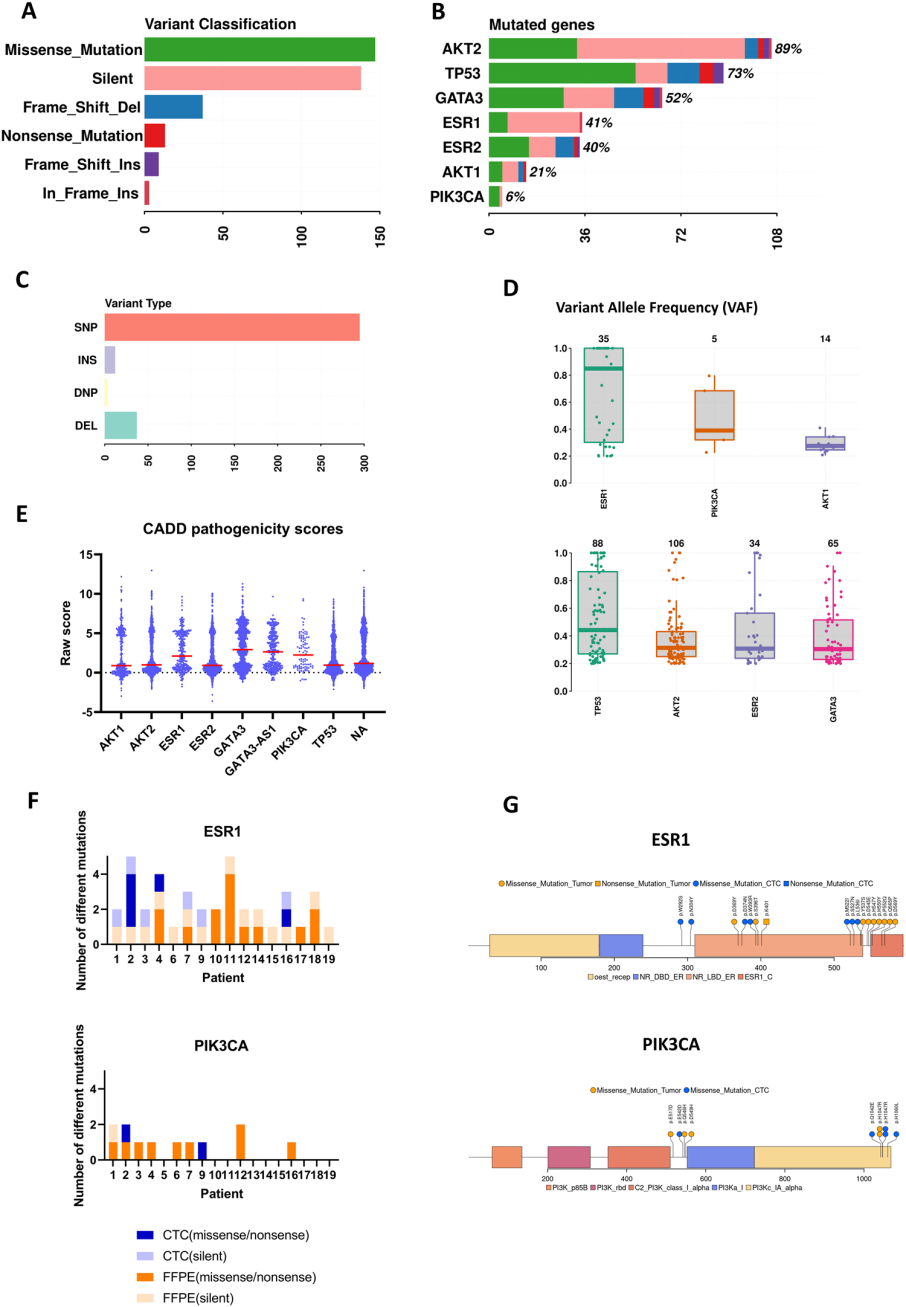
Genetic analysis of isolated single CTCs. (**A**) Variant classification of mutations detected in NGS analysis for the 7 genes. (**B**) The distribution of specific variants among all 7 analyzed genes. (**C**) Variant type analysis of detected mutations: SNP (single nucleotide polymorphism), INS (insertion), DNP (double nucleotide polymorphism), DEL (deletion) D. Variant Allele Frequency (VAF) of detected mutations; upper panel: *ESR1, PIK3CA, AKT1*, lower panel: *TP53, AKT2, ESR2, GATA3*. Box plots: interquartile range, center line: median. (**A**–**D**) Analysis performed using maftools (MAF: Mutation Annotation Format)^20^. (**E**) Pathogenicity of the detected variants estimated by Combined Annotation-Dependent Depletion (CADD) algorithm in respect to specific genes. Median marked in red F. Distribution of different mutations detected in the primary tumor and CTC of the same patient (n = 19). (**G**) Position of missense/nonsense mutations detected in primary tumor and CTC in ERS1 and PIK3CA genes.

Next Generation Sequencing (NGS) analysis was performed for single cell material amplified by Whole Genome Amplification (WGA) from 63 single cells originated from 34 different patients (Table S7). The analysis encompassed 7 genes from the estrogen signaling pathway and associated pathways: *AKT1*, *AKT2*, *ESR1*, *ESR2*, *GATA3*, *PIK3CA*, *TP53*. In total, 342 genetic changes have been identified in 60 of the 63 sequenced single cells (Fig. S2A). Most of the changes were characterized as missense mutations, followed by silent mutations (Fig. 6A). Figure 6B shows the global distribution of mutations in specific genes. A detailed representation of mutations in specific patients and their location is presented in Figs. S2 and S3.

Variant-type analysis revealed that most of the changes represented a single nucleotide polymorphism (SNP, Fig. 6C). In further analysis exonic SNPs were analyzed and mutations in noncoding regions were excluded. In total, 296 SNPs were identified in all seven genes analyzed.

NGS libraries were designed to identify mutations in hotspot regions of the *ESR1, PIK3CA* and *AKT1* genes, for the other genes the entire exonic sequence was analyzed and their analysis is presented separately (Fig. 6D). The analysis of Variant Allele Frequency (VAF) of the detected SNPs revealed that the median VAF value for most genes was between 0.2 and 0.4, suggesting that the detected variants were most probably heterozygotic or occurred in an amplified gene (Fig. 6D,E). The highest median VAF value was detected for *ESR1* (> 0.8), which is associated with the appearance of 14 variants with VAF = 1 (Fig. 6D). Interestingly, all of these variants were associated with the common populational polymorphism P325P. The polymorphism detected for the *AKT2* gene (p.R23R, T>G) has not been previously described. In the analysis, two types of variants have been detected: unique variants occurring in only one single cell and common variants occurring in at least two single cells. The unique variants were the majority of all exon SNPs, with an overall frequency of about 65%, while for the common variants, the overall frequency was about 35%. The frequency of common variants for specific genes is listed in Table S8.

Pathogenicity of the detected genetic alterations has been evaluated using Combined Annotation-Dependent Depletion (CADD) algorithm, with raw scores of specific variants, corresponding to their deleteriousness, plotted in Fig. 6E (raw data in Supplementary File S1).

#### Genetic intrapatient heterogeneity

Analysis of 39 single cells originating from material collected from 13 different patients with more than one CTC (Table S9) revealed that for 9 patients, some single CTCs share the same variants, while in 4 patients all variants identified in single CTCs were exceptional and did not occur in other cells collected from those patients (Table S10).

From a clinical perspective, considering the potential resistance to hormonal therapy, the most interesting changes were expected to occur in the *ESR1* and *PIK3CA* genes. In the *ESR1* gene 32 SNP variants were detected; the majority of the variants were silent, eight were identified as missense mutations in 8 different single CTCs (Table S11). Interestingly, missense mutations in the *ESR1* gene were detected mainly in different cells originating from the same patient. This highlights the intra-patient heterogeneity of *ESR1* missense mutations. In the *PIK3CA* gene 5 variants were identified of which 4 represented missense mutations (Table S11). For *PIK3CA* all missense mutations originated from different samples, collected from different patients.

#### Comparison of genetic changes in CTC versus primary tumor samples (FFPE)

For comparison with single-cell NGS data, we sequenced FFPE samples from 19 patients with starters specific to the *ESR1* and *PIK3CA* regions (hotspots). We chose these genes, as they are associated with resistance to anti-estrogen therapy and frequently harbor mutations.

In general, the number of different mutations in FFPE samples and CTC material did not show a previously described tendency to increase in CTCs (Fig. 6F,G, Table S12), and the PT mutations did not match the mutations in CTCs. We identified 13 different mutations in the *ESR1* gene and 4 different mutations in the *PIK3CA* gene in the FFPE samples (Table S12, Fig. 6F,G). From mutations detected in the *ESR1* gene we identified 3 silent mutations, 1 nonsense mutation, and 9 missense mutations. One FFPE sample (#11) was recognized with 5 different missense mutations in the *ESR1* gene. All mutations detected in the *PIK3CA* gene were missense mutations.

We compared the presence of mutations identified in FFPE with variants identified in the matching samples of single CTCs. The only mutations that were present in both, FFPE and the paired CTC material were the silent mutations in the *ESR1* gene (Table S12). For further analysis of changes in mutational status between FFPE and CTCs material, we focused on the missense mutations.

Four patients were identified with missense mutations in CTC-derived material, while there were no corresponding missense mutations in primary tumor samples. In one patient, the oncogenic mutation H1047R in the *PIK3CA* gene was identified in CTC, but not in the matching PT sample. One patient (#2) was identified with missense mutations that occur in single CTCs in both the *PIK3CA* and *ESR1* genes.

Interestingly, none of the missense mutations detected in the FFPE material was found in the matching CTC samples (Fig. 6G). Additionally, the molecular analysis of the FFPE samples allowed the detection of a new unreported *ESR1* genetic variant, absent from the NCBI and COSMIC databases (p.S396T, T>A, Table S11).

## Discussion

CTCs have been characterized as an independent prognostic indicator of progression-free and overall survival in the adjuvant, neoadjuvant and metastatic setting^18–20^, but are usually analyzed using the EpCAM-dependent system and with a single blood collection.

In this report we present the results of the 9 month longitudinal CTC study, with the three subsequent blood collections from patients with metastatic breast cancer. This analysis includes the evaluation of the prognostic value of monitoring the number of CTCs during treatment and the evaluation of phenotypic heterogeneity (EpCAM) and genetic heterogeneity observed in single CTCs.

### Prognostic value of CTC monitoring in mBC

High CTC counts in metastatic breast cancer were previously reported as prognostic for PFS and OS^21–24^, however, some trials fail to demonstrate the clinical utility of CTC monitoring^25^. In our study we confirmed the prognostic value of the high CTC count (≥ 5 CTCs), despite the different method of CTC detection. Furthermore, we monitored the changes in the number of CTC in individual patients during the study to establish whether the dynamics of CTC can be prognostic.

Despite the growing evidence for the clinical utility of CTCs as early recurrence markers, there is still a small number of studies that use CTC detection for consistent monitoring during treatment^9,10,26,27^. In this study we have determined that patients with an increase in CTC count from < 5CTCs to ≥ 5CTCs in the second blood collection were characterized by worse PFS and OS. Patients with persistent high CTC counts in the third collection were characterized by shorter OS. Furthermore, patients with consistently low CTC numbers (< 5CTCs) were characterized by longer PFS and OS. Additionally, we identified constant low CTC counts as a significant favorable prognostic factor for PFS and OS in the multivariable Cox model.

We also approached CTCs dynamics as a possible marker for progression. The ≥ 5CTCs count was identified as a significant marker of rapid progression in the second and third blood collections, but not in the first. This suggests that its utility as a rapid progression marker might be associated with the advancement of the disease. Furthermore, the increase in the number of CTC during treatment as a significant prognostic factor for rapid progression. The increase in CTC counts in the second blood collection and a constant increase in the number of CTCs in all collections were associated with very high odds of progression or death during 3 months from the last collection. This highlights the importance and potential utility of monitoring during therapy and supports the previous report on the utility of CTCs as an early predictor of progression^9^.

### EpCAM expression in CTC

Our EpCAM-independent approach provided a unique opportunity to compare the results with those obtained with EpCAM-dependent systems, but also enabled the analysis of EpCAM expression in a fraction of detected CTCs for which the images were of sufficiently high quality. We have identified as much as ~ 44% of EpCAM^low^ cells and ~ 0.5% of EpCAM negative cells—the population potentially omitted in EpCAM-dependent systems. In general, our data revealed a high heterogeneity of EpCAM expression in CTC not only at the interpatient but also at the intrapatient level. The tracking of changes in EpCAM status in one patient in subsequent blood collections indicated that there is no clear trend, with increase, decrease, or no change in different patients; however, the number of patients analyzed may not be sufficient to reliably conclude. Furthermore, our data indicate the difference in EpCAM status between CTC clusters and single CTCs. EpCAM expression in CTC clusters was significantly higher than in single CTCs, which may suggest that firm cell–cell junctions are crucial for cluster survival in the bloodstream.

### Genetic heterogeneity of isolated CTCs

We have characterized mutations in seven genes implicated in estrogen receptor signaling and associated pathways^28–32^, detected in isolated single CTCs.

The analysis revealed high genetic heterogeneity, with most of the changes representing missense and silent mutations that were classified as SNPs. The highest Variant Allele Frequency (VAF) was detected for the *ESR1* gene. Most of the detected changes have already been described, although we characterized a new silent mutation in the *AKT2* gene. The number, type and frequency of the identified changes suggest high genetic inter- and intrapatient heterogeneity, which is in line with previous reports^33,34^. The missense mutations characterized in the *ESR1* and *PIK3CA* genes were located primarily in the ligand binding domain and the kinase domain, respectively.

In our study, we compared the genetic material of FFPE and CTC from matching samples from 19 patients, focusing on missense mutations in the *ESR1* and *PIK3CA* genes, as the most clinically relevant. Our objective was to characterize the activating mutations that occur in CTCs possibly caused by estrogen deprivation therapy. However, the results are not easy to interpret, as none of the mutations that occurred in primary tumor samples were found in the CTC material (except silent mutations) and none of the mutations detected in CTCs were found in primary tumor samples, highlighting the high genetic heterogeneity of primary tumor and metastatic lesions. We did not observe the previously reported trend for the higher occurrence of *ESR1* and/or *PIK3CA* mutations in the CTC material compared to the FFPE samples^35^, but the number of samples in our analysis could be too low. We did observe the occurrence of new missense mutations in CTC in several cases, but only in one case it was associated with an oncogenic mutation (H1047R in the *PIK3CA* gene).

Interestingly, we detected different missense mutations in the *ESR1* gene in different CTCs that originated from the same samples. This could indicate that the analyzed CTCs originated from different metastases or from a different subclone of the same lesion. These data also support previously reported heterogeneity of CTCs in the *ESR1* gene^35,36^. We do not consider this changes as occurred during WGA process due to strict threshold value used in NGS data analysis.

In general, the high heterogeneity observed for the primary tumor and the matching CTC in both, *ESR1* and *PIK3CA*, suggests two possible conclusions: (1) that the primary tumor is genetically heterogeneous and metastatic lesions do not have missense mutations observed in PT, because they are derived from different subclones and/or (2) that dissemination could have occurred early on, before primary tumor cells acquired these specific mutations.

Furthermore, analysis of the FFPE material from the primary tumor revealed a novel mutation of *ESR1* in the LBD region, close to the E2 (or inhibitor) binding pocket (S396T). This novel mutation must be functionally tested in cell culture models to determine its effect on resistance to endocrine therapy.

## Conclusions

This report confirms the prognostic value of high CTC counts in mBC, but also presents new data on the clinical value of continuous monitoring of CTC numbers in the blood of patients with mBC. Our analysis suggests that the dynamics of CTC may be important not only for PFS and OS, but may also represent a predictor of progression in patients with mBC. Moreover, we provide data on the distribution of EpCAM expression in CTCs, which is quite unique, because most of the CTC detection systems are EpCAM-dependent. Molecular analysis of isolated CTCs points to high inter- as well as intra-patient heterogeneity. Comparative analysis of the mutations observed in CTC and matching primary tumor samples also reveal high heterogeneity, which may suggest early dissemination.

### Supplementary Information

Supplementary Information 1.

Supplementary Information 2.

## Data Availability

The datasets supporting the conclusions of this article (Next generation sequencing raw datasets) are available in the NCBI SRA repository under the accession number PRJNA1021042 (not yet released, available upon request). Corresponding author should be contacted in case of the request.

## Abbreviations

BC: Breast cancer
BSA: Bovine serum albumin
CHTH: Chemotherapy
CI: Confidence intervals
CTC: Circulating tumor cell
DAPI: 4,6-Diamidino-2-phenylindole
EpCAM: Epithelial cell adhesion molecule
ER: Estrogen receptor
FFPE: Formalin-fixed, paraffin-embedded
HER2: Human epidermal growth factor receptor 2
HR: Hazard ratio
HTH: Hormone (anti-estrogen) therapy
MAF: Mutant allele frequency
mBC: Metastatic breast cancer
NGS: Next generation sequencing
NST: No special type
OS: Overall survival
PAM50: Prediction analysis of microarray 50
pan-CK: Pan-cytokeratin
PBS: Phosphate-buffered saline
PCR: Polymerase chain reaction
PFS: Progression-free survival
PT: Primary tumor
RTH: Radiotherapy
SNP: Single nucleotide polymorphism
TNM: Tumor, nodules, metastases
VAF: Variant allele frequency
WGA: Whole genome amplification
