# Supplementary material for: Longitudinal analysis of circulating tumor cell numbers improves tracking metastatic breast cancer progression
Source: Sci Rep. 2024 Jun 5;14:12924. doi: 10.1038/s41598-024-63679-4 (PMC11153567; doi:10.1038/s41598-024-63679-4)
Supplement: Supplementary file 2 — Supplementary Information 2. [file 41598_2024_63679_MOESM2_ESM.pdf]

Figure S1

A

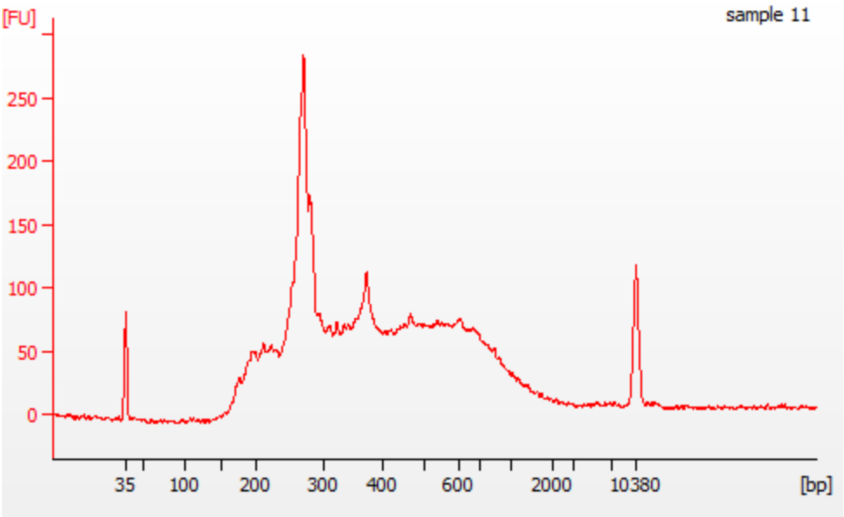

B

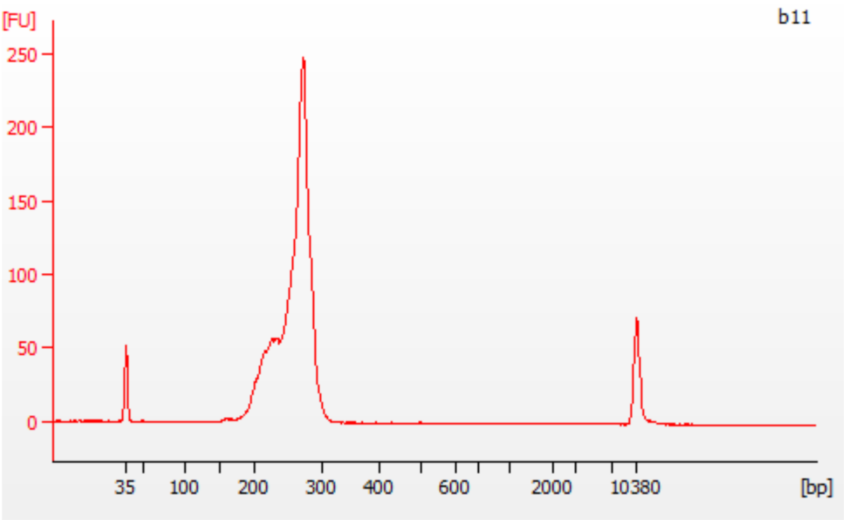

Figure S1. Results from Bioanalyzer for WGA products

A: Distribution of products above 300bp (library should be between 200-300bp) that occur as an additional peak with "bubble"; the library is not clean and not suitable for NGS analysis. B: After BluePipin purification: distribution of products between 200-3000bp, acceptable for NGS analysis,

Figure S2

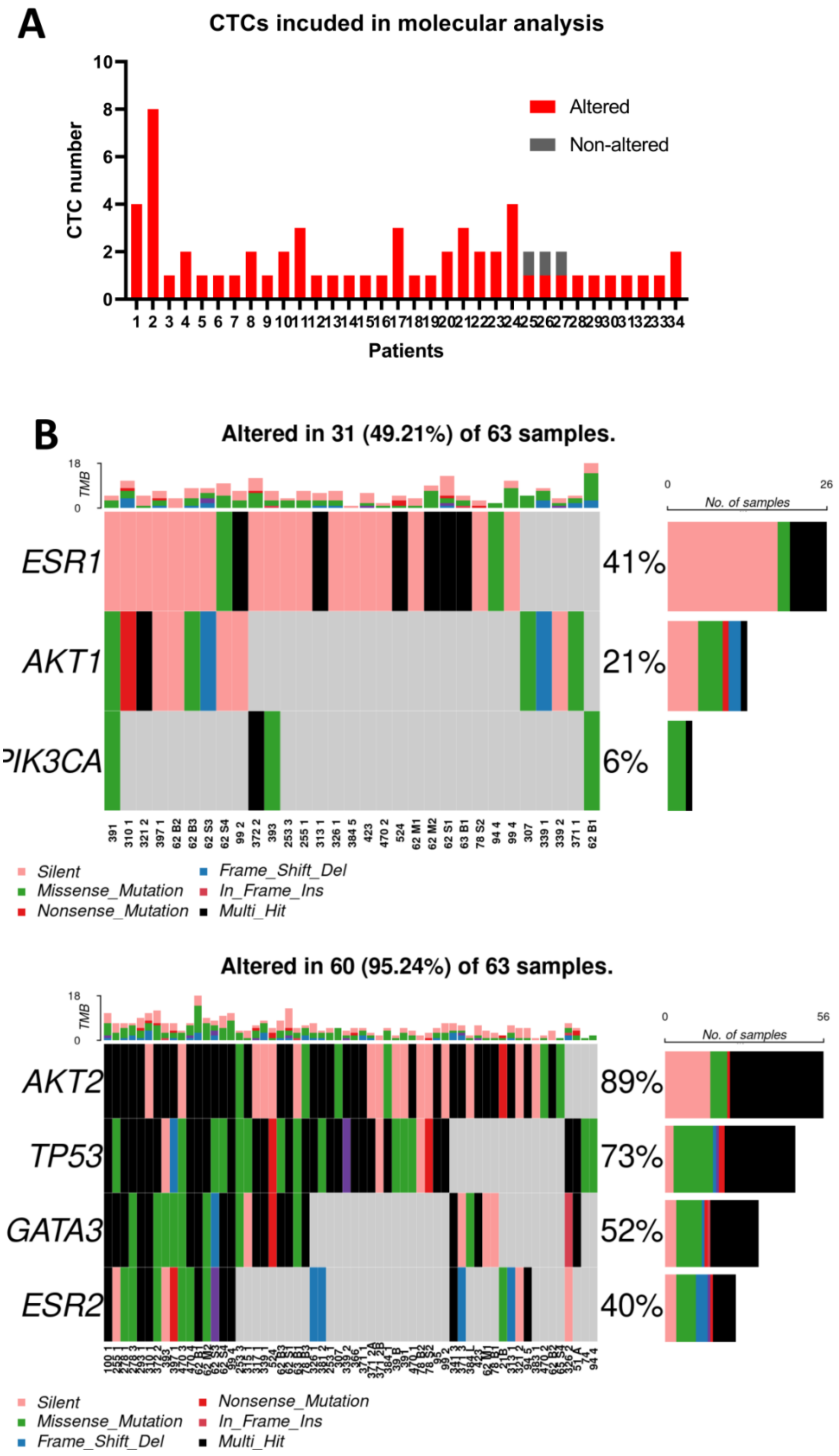

Figure S2. Genetic alternations detected in isolated CTCs

A. CTCs included in molecular analysis plotted for specific patients . In the all except three cells genetic alterations were detected B. Genetic alternations detected in CTCs presented as oncoplots. Analysis performed using maftools (MAF: Mutation Annotation Format) (20).

Figure S3

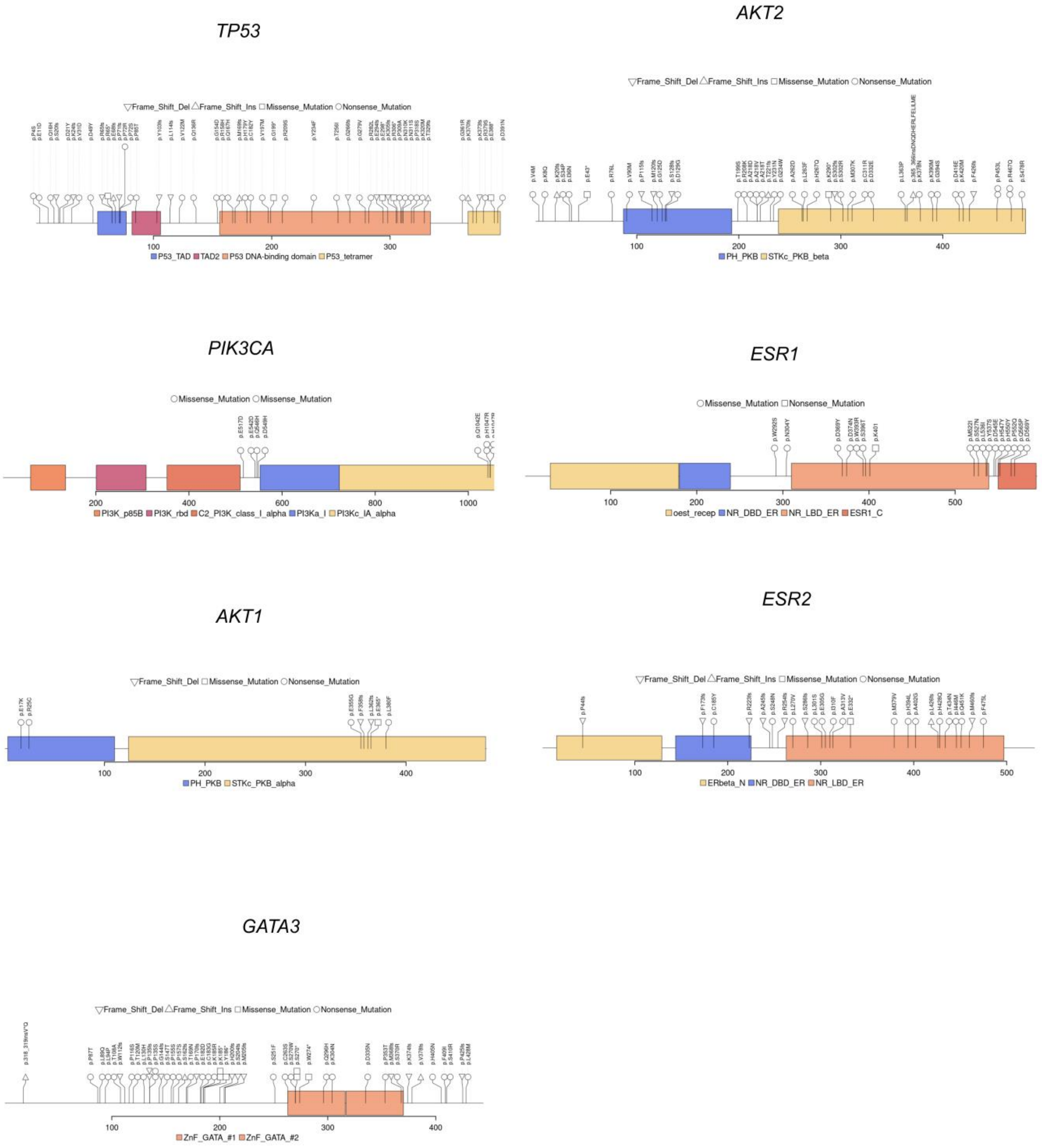

Figure S3. Distribution of mutations (all types) detected in CTCs in all 7 analyzed genes. Analysis restricted to hotspots: ESR1, PIK3CA, AKT1, whole exons: TP53, AKT2, ESR2, GATA3. Analysis performed using maftools (MAF: Mutation Annotation Format) (20).

Table S1. Conditions forSanger sequencing of FFPE material

PCR Stage	Temperature	Time	No. of cycles
Initial Denaturation	95°C	10min	-
Denaturation	95°C	15s	×40
Primer Hybridization	55°C	30s	
Elongation	72°C	30s	
Final Elongation	72°C	5min	-
Storage	4°C	∞	-

Table S2. Primers used for amplification of the specific regions from *ESR1* and *PIK3CA* genes.

Gene	Forward primer	Reverse primer	Product length
ESR1 8 exon	5’- TCTGTGTCTTCCCACCTACAGT-3’	5’- ATGCGATGAAGTAGAGCCCG-3’	200bp
ESR1 5 exon	5’- GCTTTGTGGATTTGACCCTCCA-3’	5’- AGAGCAAGTTAGGAGCAAACAG-3’	135bp
ESR1 4 exon	5’-TGAAACACAAGCGCCAGAGA-3’	5’- CCAGGTTGGTCAGTAAGCCC-3’	255bp
PIK3CA 9 exon	5’- AGCTAGAGACAATGAATTAAGGGA -3’	5’- TCCATTTTAGCACTTACCTGTGAC -3’	130bp
PIK3CA 20 exon	5’- AACTGAGCAAGAGGCTTTGGA -3’	5’- CAATCGGTCTTTGCCTGCTG -3’	200bp

Table S3. Reference groups in KM analysis (bold)

1st collection: <5CTCs

Group	Median PFS	Median OS
**<5 CTCs**	**9.83**	**N/A**
>5CTCs	6.3	11.33

2nd collection: <5 CTCs, no change, constant <5 CTCs

Group	Median PFS	Median OS
**<5 CTCs**	**10.7**	**N/A**
>5CTCs	3.3	8.85
**No change**	**16.98**	**N/A**
Rise	4.47	15.95
Lost	9.5	18.3
**1: <5; 2: <5**	**11**	**N/A**
1: <5; 2: >5	3.06	9.67
1: >5; 2: >5	3.3	8.03

3rd collection: <5 CTCs, no change, constant <5 CTCs

Group	Median PFS	Median OS
**<5 CTCs**	**N/A**	**N/A**
>5CTCs	8.17	15.78
**No change**	**N/A**	**N/A**
Rise	11.6	N/A
Lost	9.6	N/A
**1: <5; 2: <5; 3: <5**	**N/A**	**N/A**
1: <5; 2: <5; >5	7.05	N/A
1: <5; 2: >5; >5	7.9	8.17
1: >5; 2: >5; 3: >5	N/A	4.9

TableS4. The clinical and pathological data with reference levels used for multivariable model. The cut-off value for age variable was set as the median value in the patients' cohort.

Variable	Refrence level	Other levels
Histopatological subtype	NST	LOBULARE<br>OTHER<br>DUCTALE-LOBULARE
RTH during observation	No RTH during observation	RTH during observation
TNM status	T_XN0M0	T_XN_XM0<br>$T_XN_XM_X$
Type of treatment	HTH	HTH + CDK4/6 inhibitors<br>HTH + CHTH
Age	<65	≥65

Table S5. Detailed results of Cox proportional hazard regression models.

	OS		p-value	PFS		p-value
	HR	95% CI		HR	95% CI	
1st collection						
Univariable						
≥5 CTCs	2.3	1.201 - 4.083	0.0071	1.672	0.9277 - 2.806	0.0668
Multivariable						
≥5 CTCs	2.323	1.175 - 4.320	0.0105	1.987	1.067 - 3.493	0.0223
2nd collection landmark analysis						
Univariable						
≥5 CTCs	2.724	1.370 - 5.010	0.0022	2.539	1.421 - 4.263	0.0008
Constant ≥5 CTCs	1.834	0.4461 - 4.986	0.3076	2.147	0.6531 - 5.188	0.1387
Constant <5 CTCs	0.3686	0.2126 - 0.6633	0.005	0.4813	0.3046 - 0.7893	0.0025
Increase from <5 CTCs to ≥5 CTC	2.933	1.340 - 5.723	0.0033	2.516	1.298 - 4.457	0.0031
Decrease from ≥5 CTCs to <5 CTCs	1.943	0.7439 - 4.197	0.1257	1.265	0.5306 - 2.545	0.5508
General increase in CTCs number	1.806	1.026 - 3.087	0.0343	2.174	1.383 - 3.342	0.0005
General decrease in CTCs number	1.173	0.6181 - 2.094	0.6047	1.057	0.6391 - 1.680	0.8214
Multivariable						
≥5 CTCs	3.004	1.453 - 6.016	0.002	2.359	1.296 - 4.051	0.003
Constant ≥5 CTCs	2.086	0.4738 - 6.426	0.5243	2.377	0.6461 - 6.904	0.1437
Constant <5 CTCs	0.3338	0.1798 - 0.6334	0.0006	0.4423	0.2717 - 0.7429	0.0014
Increase from <5 CTCs to ≥5 CTC	3.067	1.343 - 6.348	0.0042	2.261	1.149 - 4.100	0.0112
Decrease from ≥5 CTCs to <5 CTCs	1.869	0.6958 - 4.237	0.1671	1.684	0.6696 - 3.667	0.2231
General increase in CTCs number	1.571	0.8631 - 2.800	0.1301	1.99	1.247 - 3.116	0.0031
General decrease in CTCs number	1.183	0.6160 - 2.144	0.5952	1.163	0.6917 - 1.882	0.553
3rd collection landmark						
Univariable						
≥5 CTCs	1.661	0.8648 - 3.067	0.1129	1.559	0.9543 - 2.476	0.0667
Constant ≥5 CTCs	3.296	0.5355 - 10.79	0.1008	0.8709	0.2123 - 2.354	0.8156
Constant <5 CTCs	0.541	0.2969 - 0.9941	0.0447	0.6245	0.4011 - 0.9838	0.0389
Persistent ≥5CTCs from 2nd collection	3.728	1.112 - 9.346	0.0128	1.772	0.6215 - 3.965	0.2164
Increase to ≥5CTCs only in 3rd collction	0.8898	0.3627 - 1.880	0.7774	1.522	0.8334 - 2.603	0.1455
General increase	1.959	0.7428 - 4.309	0.1268	1.275	0.6135 - 2.377	0.4774

in CTCs number in 2nd and 3rd collection (constant increase)						
General decrease in CTCs numbers in 2nd and 3rd collection (constant decrease)	1.63	0.3942 - 4.485	0.4148	1.92	0.5798 - 4.707	0.2097
Multivariable						
≥5 CTCs	2.29	1.145 - 4.417	0.0154	1.72	1.027 - 2.804	0.0336
Constant ≥5 CTCs	3.279	0.4968 - 12.54	0.1298	0.9485	0.2225 - 2.771	0.9322
Constant <5 CTCs	0.4434	0.2246 - 0.8725	0.0181	0.5867	0.3671 - 0.9468	0.0267
Persistent ≥5CTCs from 2nd collection	7.001	1.744 - 23.33	0.0027	2.251	0.7468 - 5.552	0.1054
Increase to ≥5CTCs only in 3rd collction	1.157	0.4568 - 2.552	0.7354	1.55	0.8211 - 2.751	0.1529
General increase in CTCs number in 2nd and 3rd collection (constant increase)	1.929	0.7056 - 4.513	0.1574	1.45	0.6878 - 2.753	0.2887
General decrease in CTCs numbers in 2nd and 3rd collection (constant decrease)	1.233	0.2844 - 3.703	0.7409	1.605	0.4743 - 4.089	0.3767

Table S6. Results of logistic regression with data from CTCs analysis as predictor for death or progression occurring in 3 months from collection.

	Progression or death occuring within 3 months from the collection		
	OR	95% CI	p-value
1st collection			
≥5 CTCs	1.721	0.5070 – 5.161	0.3634
2nd collection			
≥5 CTCs	6.143	2.111 - 18.88	0.0009
Increase to ≥5 CTCs	5.359	1.571 - 19.61	0.0078
Constant <5 CTCs	0.2437	0.09441 - 0.6230	0.0034
Constant ≥5 CTCs	5.056	0.7995 - 39.89	0.0832
3rd collection			
≥5 CTCs	3.273	1.088 - 9.916	0.0352
Persistnet ≥5CTCS from 2nd collection	4.333	0.5358 - 28.45	0.152
Increase to ≥5 CTCs inly in 3rd collection	2.597	0.7293 - 8.396	0.1341
Constant <5 CTCs	0.3646	0.1197 - 1.069	0.0657
Constant ≥5 CTCs	3.067	0.1376 - 33.99	0.4063
General increase in CTCs number in 2nd and 3rd collection (constant increase)	8.091	1.976 - 33.75	0.0045

Table S7. Cells used in molecular analysis, with the indication of patient and collection number

patient	no. of cells in NGS	No of cells from 1st collection	No of cells from 2nd collection	No of cells from 3rd collection
1	1*	1		
2	1	1		
3	1	1		
4	1	1		
5	1	1		
6	1	1		
7	1	1		
8	1	1		
9	1		1	
10	1		1	
11	1		1	
12	1		1	
13	1		1	
14	1		1	
15	1		1	
16	1		1	
17	1		1	
18	1			1
19	1			1
20	2	1		1
21	2	2		
22	2		2	
23	2	1		1
24	2		1	1
25	2	2		
26	2	2		
27	2	2		
28	2			2
29	3*			3
30	3	3		
31	3	1	2	
32	4*		4	
33	4		3	
34	4			4
35	8	8		

*one cell eliminated from the analysis

Table S8. Mutations detected in more than one single cell and their frequency for specific gene.

Gene	Mutation	No. of cells	Frequency
AKT1	p.H354H	4	6,45%
AKT2	p.K111K	2	3,23%
AKT2	p.S398S	2	3,23%
AKT2	p.P453L	2	3,23%
AKT2	p.R467Q/F	2	3,23%
AKT2	p.R23R	42	67,74%
ESR1	p.K531K	2	3,23%
ESR1	p.N532N	2	3,23%
ESR1	p.P325P	16	25,80%
ESR2	p.V328V	2	3,23%
GATA3	p.P135S/A/S	2	3,23%
GATA3	p.T418T	3	4,83%
TP53	p.C182Y	2	3,23%
TP53	p.P4S	3	4,83%
TP53	p.P72R	23	37,10%

Table S9. Numbers of analyzed single CTCs originated from one patient.

**Patient no.**	**No. of CTCs**
1	4
2	8
4	2
8	2
10	2
11	3
17	3
20	2
21	3
22	2
23	2
24	4
34	2

Table S10. Variants detected in at least two cells originated from one patient.

**Gene**	**Patient no.**	**Mutation**	**Variant Type**	**No. of cells with variant/No. of cells analyzed**
AKT2	1	p.R23R	Silent	3/4
AKT2	2	p.R23R	Silent	8/8
ESR1	2	p.P325P	Silent	3/8
TP53	2	p.P72R	Missense	4/8
AKT2	8	p.R23R	Silent	2/2
TP53	10	p.P72R	Missense	2/2
AKT2	11	p.R23R	Silent	2/3
TP53	20	p.P72R	Missense	2/2
GATA3	23	p.T418T	Silent	2/2
AKT2	24	p.R23R	Silent	3/4
AKT2	34	p.R23R	Silent	2/2

Table S11. Missense mutations detected in *ESR1* and *PIK3CA* genes.

ESR1	
Mutation	Patient no.
p.L536I	2
p.M522I	2
p.D374N	2
p.S527N	4
p.Q375E	4
p.N304Y	16
p.W292S	16
p.W393R	33
PIK3CA	
Mutation	Patient no.
p.Q1042E	2
p.H1047R	9
p.E542D	34
p.H1060L	28

Table S12. Mutations detected in primary tumor FFPE samples and the matching CTCs in *ESR1* and *PIK3CA* genes. Missense/Nonsense mutations – light grey, silent mutations - white

Patient no.	Gene	Mutation	FFPE	CTC
1	ESR1	p.P325P	+	+
	PIK3CA	p.H1047R	+	-
2	ESR1	p.P325P	+	+
	ESR1	p.L536I	-	+
	ESR1	p.D374N	-	+
	ESR1	p.M522I	-	+
	PIK3CA	p.E517D	+	-
	PIK3CA	p.Q1042E	-	+
3	ESR1	p.P325P	+	+
	PIK3CA	p.E517D	+	-
4	ESR1	p.S527N	-	+
	ESR1	p.D369Y	+	-
	ESR1	p.K401*	+	-
	ESR1	p.P325P	+	-
	PIK3CA	p.E517D	+	-
5	ESR1	p.P325P	+	-
6	ESR1	p.P325P	+	+
	PIK3CA	p.D549H	+	-
7	ESR1	p.P325P	+	+
	ESR1	p.D569Y	+	-
	PIK3CA	p.Q546H	+	-
9	ESR1	p.P325P	+	+
	PIK3CA	p.H1047R	-	+
10	ESR1	p.K401*	+	-
	ESR1	p.E517D	+	-
11	ESR1	p.P325P	+	-
	ESR1	p.R548R	+	-
	ESR1	p.H547Y	+	-
	ESR1	p.H550Y	+	-
	ESR1	p.P552Q	+	-
	ESR1	p.S559S	+	-
	ESR1	p.Q565P	+	-
12	ESR1	p.P325P	+	-
	ESR1	p.D545E	+	-
	PIK3CA	p.E517D	+	-
	PIK3CA	p.D549H	+	-
13	ESR1	p.P325P	+	-
14	ESR1	p.S396T	+	-
15	ESR1	p.P325P	+	-
16	ESR1	p.P325P	+	+
	ESR1	p.W292S	-	+
	PIK3CA	p.D549H	+	-

17	*ESR1*	p.S396T	+	-
18	*ESR1*	p.P325P	+	-
	ESR1	p.K401*	+	-
	ESR1	p.Q565P	+	-
19	*ESR1*	p.P325P	+	-
